# Association between Increased Gastric Juice Acidity and Sliding Hiatal Hernia Development in Humans

**DOI:** 10.1371/journal.pone.0170416

**Published:** 2017-01-20

**Authors:** Hiroshi Kishikawa, Kayoko Kimura, Asako Ito, Kyoko Arahata, Sakiko Takarabe, Shogo Kaida, Takanori Kanai, Soichiro Miura, Jiro Nishida

**Affiliations:** 1 Department of Gastroenterology, Tokyo Dental College, Ichikawa General Hospital, Ichikawa, Chiba, Japan; 2 Department of Internal Medicine, Division of Gastroenterology and Hepatology, Keio University, Shinjyuku-ku, Tokyo, Japan; 3 Department of Internal Medicine, Division of Gastroenterology and Hepatology, Keio University, Shinjyuku-ku, Tokyo, Japan; Universitatsklinikum Aachen, GERMANY

## Abstract

**Objectives:**

Several clinical factors; overweight, male gender and increasing age, have been implicated as the etiology of hiatal hernia. Esophageal shortening due to acid perfusion in the lower esophagus has been suggested as the etiological mechanism. However, little is known about the correlation between gastric acidity and sliding hiatus hernia formation. This study examined whether increased gastric acid secretion is associated with an endoscopic diagnosis of hiatal hernia.

**Methods:**

A total of 286 consecutive asymptomatic patients (64 were diagnosed as having a hiatal hernia) who underwent upper gastrointestinal endoscopy were studied. Clinical findings including fasting gastric juice pH as an indicator of acid secretion, age, sex, body mass index, and *Helicobacter pylori* infection status determined by both *Helicobacter pylori* serology and pepsinogen status, were evaluated to identify predictors in subjects with hiatal hernia.

**Results:**

Male gender, obesity with a body mass index >25, and fasting gastric juice pH were significantly different between subjects with and without hiatal hernia. The cut-off point of fasting gastric juice pH determined by receiver operating curve analysis was 2.1. Multivariate regression analyses using these variables, and age, which is known to be associated with hiatal hernia, revealed that increased gastric acid secretion with fasting gastric juice pH <2.1 (OR = 2.60, 95% CI: 1.38–4.90) was independently associated with hiatal hernia. Moreover, previously reported risk factors including male gender (OR = 2.32, 95% CI: 1.23–4.35), body mass index >25 (OR = 3.49, 95% CI: 1.77–6.91) and age >65 years (OR = 1.86, 95% CI: 1.00–3.45), were also significantly associated with hiatal hernia.

**Conclusions:**

This study suggests that increased gastric acid secretion independently induces the development of hiatal hernia in humans. These results are in accordance with the previously reported hypothesis that high gastric acid itself induces hiatal hernia development.

## Introduction

Hiatal hernia is a herniation of the gastric cardia through the esophageal hiatus of the diaphragm. It is differentiated into four types (types I-IV), of which type I (sliding hiatal hernia), characterized by both widening of the muscular hiatal aperture of the diaphragm and laxity of the phreno-esophageal membrane, accounts for 95% of cases [1]. The association between sliding hiatal hernia and gastroesophageal reflux disease (GERD) has long been recognized because of the high prevalence of their coexistence [2]. Hiatal hernia reduces lower esophageal sphincter tone, which results in a loss of the pinchcock effect of prevention of gastric acid reflux, and also acts as an acid reservoir that allows ready access of gastric juice into the esophagus, thus contributing to prolonged esophageal acid exposure leading to GERD [3, 4].

Two traditional etiologies of hiatal hernia have been suggested: decreased elasticity of ligamentous structures around the diaphragmatic hiatus due to increased age, and esophageal axial pressure strain through the diaphragm due to increased intragastric pressure induced mainly by obesity [5, 6]. A meta-analysis of risk factors of hiatal hernia reported by Menon et al. suggested that the prevalence of hiatal hernia increases with age, increasing body mass index, and male sex, which is consistent with the traditional explanation of the pathogenesis of hiatal hernia [7]. Esophageal shortening has been regarded as the other important factor in hiatal hernia development. Several investigators have reported that acid perfusion in the esophagus results in reflex contraction of the esophageal longitudinal smooth muscle and consequent esophageal shortening in both experimental animal studies and humans. Paterson et al. suggested that luminal acid in the lower esophagus activates mast cell degranulation and activation of capsaicin-sensitive neurokinin neurons, which contribute to sustained contraction of the esophageal longitudinal smooth muscle [8, 9]. A report from Japan by Iwakiri et al. noted that the prevalence of hiatal hernia in patients with closed-type (low-grade) gastric mucosal atrophy was significantly higher than that of open-type (high-grade) gastric mucosal atrophy, suggesting that acid exposure to the esophagus induces hiatal hernia formation clinically [10]. It is possible that acid-induced esophageal shortening may result in formation of a vicious cycle, whereby the hiatal hernia exacerbates reflux, which in turn induces more esophageal shortening and the production of a larger hernia.

Based on these findings, increased gastric acid may cause esophageal shortening and hiatal hernia, although little is known about the direct correlation between gastric acidity itself and sliding hiatal hernia formation in humans.

The aims of this study were to determine the clinical factors associated with endoscopic hiatal hernia and especially whether low gastric pH predisposes to hiatal hernia development.

## Materials and Methods

### Study population

Between 2007 and 2014, 742 consecutive subjects, aged 21 to 86 years, who attended Tokyo Dental College Ichikawa General Hospital outpatient clinic for routine upper gastrointestinal endoscopy were prospectively enrolled. Gastrointestinal endoscopy was performed using electrical panendoscopes (types XQ260, Olympus, Tokyo, Japan). All endoscopies were performed by a single experienced gastroenterologist (HK). Exclusion criteria were as follows: 1) use of histamine-2 receptor antagonists or proton pump inhibitors within the preceding month; 2) use of *H*. *pylori* eradication therapy before the study; 3) esophageal or gastric cancer, a past history of these cancers, or any kind of esophageal or gastric surgery; 4) presence of viral diseases, such as acute respiratory diseases; 5) pregnancy or lactation; and 6) a history of severe renal and/or liver dysfunction.

Sliding hiatal hernia is assessed by endoscopy, although there is little global uniformity with respect to the endoscopic diagnosis of sliding hernia [11]. In this study, sliding hiatal hernia was diagnosed when anatomic disruption of the esophago -columnar junction was over the diameter of the shaft of the endoscope, as seen from a retroflexed endoscopic view of the cardia. Esophageal mucosal breaks with esophagitis were graded according to the Los Angeles Classification of Esophagitis [12]. Reflux esophagitis was diagnosed when endoscopic findings of reflux esophagitis were grade A (mucosal breaks no longer than 5-mm) or higher. This study was approved by the Tokyo Dental College Ichikawa General Hospital Ethics Committee and was conducted according to the principles of the Second Declaration of Helsinki. All patients provided their written, informed consent prior to enrollment.

### Assays for antibodies against H. pylori and for serum pepsinogen-I and pepsinogen-II

Measurement of serum pepsinogen-I, pepsinogen-II, and *H*. *pylori* IgG antibodies was contracted out to LSI Medience Co., Ltd. (Tokyo, Japan) as described previously [13, 14]. Subjects were defined as “*H*. *pylori* negative” when the criteria of normal pepsinogen status (pepsinogen-I > 70 ng/mL or pepsinogen-I/II ratio > 3) and serum *H*. *pylori* antibody titer of < 3 U/ml were satisfied, based on previous reports, including our own, suggesting that the combination of normal pepsinogen levels and *H*. *pylori* antibody titer <3 U/ml is a useful predictor of “true negative” *H*. *pylori* cases [15–18].

### pH measurement

Gastric acid secretion was evaluated using fasting gastric pH, a reliable indicator of acid secretion and acidity of gastric juice). Gastric juice samples were collected through a sterile tube during endoscopy, and their pH was measured with a glass electrode as described previously [13, 14, 16].

### Statistical analysis

Statistical analysis was performed with the software program Statistical Package for Social Sciences (SPSS) v 22 (IBM, Chicago, IL, USA) for Windows. Continuous data were summarized as median (25th and 75th percentiles), and counts and percentages were used for categorical variables. Comparisons of continuous variables between hiatal hernia cases and those without hiatal hernia were performed using the Mann-Whitney U test. Chi-square or Fisher’s exact test was used for two-group comparisons for categorical variables. A two-tailed p value was considered significant when its values was <0.05. We used receiver operating characteristic (ROC) curves analysis to determine the optimal cutoff values for fasting gastric juice pH in the diagnosis of hiatal hernia cases. A multivariate logistic regression analysis yielding odds ratios (ORs) and 95% confidence intervals (CIs) was performed to identify predictors of hiatal hernia using factors that were found to be associated with hiatal hernia in univariate analysis (*p*< 0.05), and age, which is known to be associated with hiatal hernia formation. Inclusion of variables was assessed using a stepwise selection method, with entry criteria of *p* ≤ 0.05 and removal criteria of *p* > 0.10, which was used to establish the final multivariate predictive model.

## Results

### Study participants

**Fig 1:**
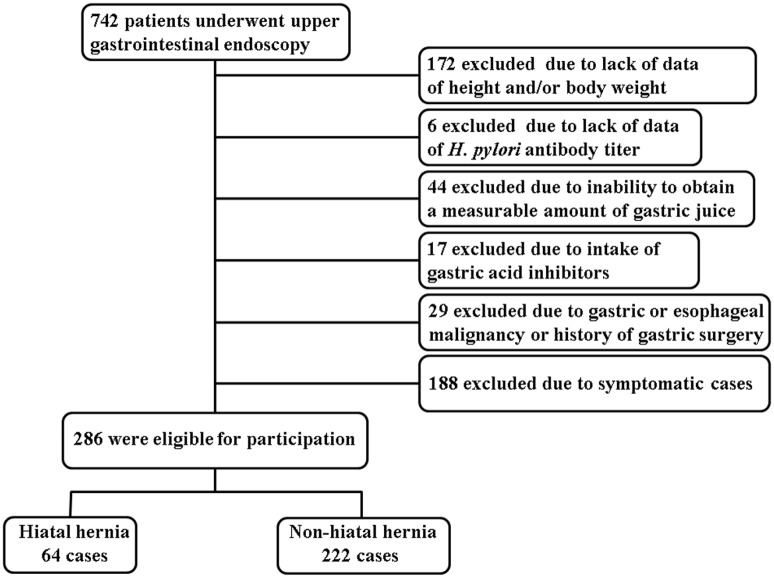
Flow diagram of study populations.

A total of 742 subjects were initially enrolled in the study. Patients with a lack of the data on height and/or body weight (n = 172), a lack of the data on *H*. *pylori* antibody titer (n = 6), insufficient gastric juice specimen to measure pH (n = 44), intake of proton pump inhibitor or H2-receptor antagonist (n = 17), gastric or esophageal malignancy or history of prior gastric surgery (n = 29), or symptomatic cases (n = 188) were excluded, as shown in Fig 1. A total of 286 subjects were included for the final analysis. Based on the endoscopic findings, they were divided into two groups: hiatal hernia (n = 64) and non- hiatal hernia cases (n = 222). The reasons for performing endoscopy were as follows; abnormalities on barium X-ray (n = 111, 38.8%), screening purpose in asymptomatic individuals (n = 105, 36.7%), annual endoscopic follow-up (n = 48, 16.8%), and positive results on ABC gastric cancer screening (n = 22, 7.7%).

### Patients’ characteristics

The characteristics of the study population are shown in Table 1 and S1 Table. A total of 286 subjects (158 men (55.2%)) met the study inclusion criteria. Median subjects age was 65.0 years and the prevalence of aged subjects (>65 years) was 48.3%. Median BMI was 22.8 kg/m^2^, and 55 (19.2%) subjects were considered obese due to a BMI of >25kg/m^2^ according to the criteria of The Japanese Society for the Study of Obesity [19]. One hundred and fourteen (39.9%) subjects were *H*. *pylori* negative, defined by the combination of *H*. *pylori* serology and serum pepsinogen levels, and the median fasting gastric juice pH was 1.97. Overall, 64 (22.4%) subjects had hiatal hernia.

**Table 1:** Characteristics of the study population (N = 286).

**Age (years)**	
** ≤65**	**148 (51.7%)**
** >65**	**138 (48.3%)**
** Median**	**65.0 (57.0, 72.0)**
**Male Sex (%)**	**158 (55.2%)**
**BMI (kg/m**^**2**^**)**	
** ≤25**	**231 (80.8%)**
** >25**	**55 (19.2%)**
** Median**	**22.8 (20.6, 24.5)**
**Peptic ulcer and ulcer scar (%)**	**35 (12.2%)**
***H*. *pylori* negative cases (%)**	**114 (39.9%)**
**Hiatal hernia positive (%)**	**64 (22.4%)**
**Reflux esophagitis positive (%)**	**36 (12.6%)**
**Fasting gastric juice pH**	
** Median**	**1.97 (1.54, 5.06)**

Values are presented as Median (25th and 75th percentiles) or N (%). BMI, Body mass index.

### Comparison of variables between subjects with and without hiatal hernia

Table 2 shows the demographic and clinical characteristics of the study subjects based on the presence of endoscopic hiatal hernia.

**Table 2:** Characteristics of the subjects with and without hiatal hernia and univariate analysis of risk factors for hiatal hernia.

Characteristic	No hiatal hernia cases	Hiatal hernia cases	p value
**Age (years) >65**	**104/222 (46.8%)**	**34/64 (53.1%)**	**0.4**
** median**	**65.0 (57.0, 71.3)**	**66.5 (56.3, 72.0)**	**0.95**
**Sex Male**	**112/222 (50.5%)**	**46/64 (71.9%)**	<0.01*
**BMI (kg/m2) >25**	**32/222 (14.4%)**	**23/64 (35.9%)**	<0.001*
** Median**	**22.5 (20.4, 24.1)**	**24.1 (22.2, 25.6)**	<0.001*
***H*. *pylori* seronegative**	**85/222 (38.3%)**	**29/64 (45.3%)**	**0.32**
**Reflux esophagitis >Grade A**	**22/222 (9.9%)**	**14/64 (21.9%)**	<0.05*
**Fasting gastric pH**			
** Median**	**2.04 (1.56, 5.98)**	**1.79 (1.44, 2.80)**	<0.05*

Prevalence of male gender (*p*<0.01), obesity with a BMI>25 (*p*<0.001), and reflux esophagitis (*p*<0.05) were significantly higher in subjects with hiatal hernia. Fasting gastric juice pH (low fasting gastric juice pH indicating less mucosal atrophy with preserved acid secretion) was significantly lower (*p*<0.05) in subjects with hiatal hernia. Statistics are presented are Median (25th and 75th percentiles) or N (%). BMI, Body mass index.

* A p-value of less than 0.05 was considered statistically significant.

Patient age (median 65.0 years), older age distribution (>65 years) and prevalence of *H*. *pylori*-negative subjects did not differ between subjects with and without hiatal hernia. However, the median levels of BMI, and the percentage of BMI >25 kg/m^2^ subjects were significantly higher in the hiatal hernia group compared to non-hiatal hernia group. The frequencies of male sex, and reflux esophagitis were also higher among patients with hiatal hernia than in those without hiatal hernia. Median fasting gastric juice pH was significantly lower in subjects with hiatal hernia.

### Predictive value of fasting gastric juice pH for hiatal hernia

**Fig 2:**
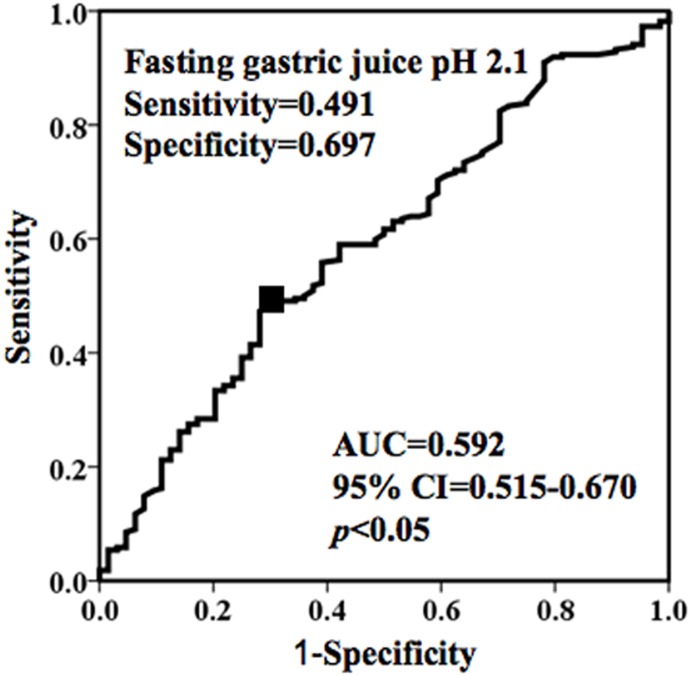
Receiver operating characteristic (ROC) curve analysis using fasting gastric juice pH as a predictor of hiatal hernia. At a cut-off value of 2.1, gastric juice pH exhibited 49.1% sensitivity and 69.7% specificity for predicting hiatal hernia, and area under the curve (AUC) of 0.592 (95% CI 0.515–0.670, *p*<0.05).

Using receiver operating characteristic (ROC) curve analysis to determine the optimal fasting gastric juice pH cutoff values for predicting hiatal hernia subjects, a pH of 2.1 showed the most accuracy with a total sensitivity and specificity of 1.19 (Fig 2). Furthermore, the positive predictive value (PPV) and negative predictive value (NPV) of this cutoff value was 0.27 and 0.85, respectively, for the prediction of hiatal hernia subjects.

### Factors associated with hiatal hernia on multivariate logistic analysis

Stepwise multiple logistic regression analysis was performed to identify predictors of a sliding hiatal hernia using factors identified on univariate analysis as significant (sex, elevated BMI, and low fasting gastric juice pH) and increasing age, which is an established risk factor for hiatal hernia development. For the fasting gastric juice pH, a value of ≤ 2.1 was chosen as a covariate based on ROC curve analysis.

Multivariate regression analysis showed that increased gastric acid secretion with fasting gastric juice pH≤ 2.1 (OR, 2.60; 95% CI, 1.38–4.90) was independently associated with hiatal hernia development. The previously reported risk factors of male gender (OR, 2.32; 95% CI, 1.23–4.35), BMI >25 (OR, 3.49; 95% CI, 1.77–6.91), and age >65 years (OR, 1.86; 95% CI, 1.00–3.45) were also shown to be predictors of hiatal hernia (Table 3).

**Table 3:** Multivariate analysis: Relationship of hiatal hernia with fasting gastric juice pH, sex, body mass index, *H*. *pylori* infection status and age.

Characteristic	Standardized coefficient	Odds ratio	95% CI		Significance
			Lower	Upper	
**Fasting gastric pH ≤2.1**	**0.96**	**2.60**	**1.38**	**4.90**	<0.01*
**(reference: pH >2.1)**					
**Male sex**	**0.84**	**2.32**	**1.23**	**4.35**	<0.01*
**(reference: female)**					
**BMI >25**	**1.25**	**3.49**	**1.77**	**6.91**	<0.001*
**(reference: ≤25 kg/m**^**2**^**)**					
**Age >65 y**	**0.62**	**1.86**	**1**	**3.45**	**0.05**
**(reference: ≤65 y)**					

Stepwise multivariate logistic regression analyses incorporating sex, increase in BMI, low fasting gastric juice pH and increasing age were performed to explore the independent relationships of these factors with hiatal hernia. Fasting gastric juice pH ≤2.1 was a statistically significant predictor of hiatal hernia, indicating that increased gastric acid secretion is significantly associated with an increased risk of hiatal hernia. The previously reported risk factors of male gender, BMI >25 kg/m^2^, and older age of >65 years were also shown to be predictors of hiatal hernia. BMI, body mass index; CI, confidence interval.

* A p-value of less than 0.05 was considered significant.

## Discussion

In the present study, there was a significant positive association between endoscopic hiatal hernia development and high gastric acid secretion. Several reported risk factors for hiatal hernia, such as male sex, obesity, and increasing age, were also shown to be predictors of hiatal hernia in the present study [7, 20]. Several previous studies indicated that acid injury of the esophageal mucosa causes longitudinal muscle contraction and esophageal shortening, which induces the development of hiatal hernia [8, 9]. However, to date, it has not been clarified whether high gastric acid secretion, which causes increased acid exposure to the lower esophagus, is associated with hiatal hernia in humans. Thus, this is the first clinical study suggesting that high acid secretion itself may induce hiatal hernia development in humans, based on a direct evaluation of the association between acid secretory function and endoscopic findings of hiatal hernia in over 400 subjects.

In the authors’ view, measuring fasting gastric pH is comparable to performing a pH study, although a pH study is important and should be performed in future studies to elucidate the etiology of hiatal hernia. Two reasons can justify the use of fasting gastric juice pH as an indicator of the degree of acid reflux to the esophagus, as well as of acid secretory function. Fasting gastric juice pH has been reported to correlate well with acid secretory function (basal acid output) [21]; hence, the present results directly explain the previous reports by Iwakiri et al [10], suggesting the correlation between hiatal hernia and hyperacidity predicted by endoscopic atrophy. Meanwhile, the lower esophagus is physiologically exposed to gastric acid even in normal subjects (normal range in a pH study for pH 4 holding time is within 8.42% in the supine position); thus, high gastric acid itself affects the environment of the gastroesophageal junction even without abnormal reflux [22]. Recently, special attention has been given to the significance of measuring fasting gastric pH. Ayazi et al. reported a significant inverse, dose-dependent relationship between gastric pH and the degree of esophageal acid exposure [23]. Based on their report, higher the acidity of gastric juice, greater the exposure of the esophagus to the gastric acid. Hence, the present results are compatible with the previous reports suggesting the correlation between hiatal hernia and experimental lower esophageal acid exposure.

Clinically, a diagnosis of esophageal shortening is not important, except in the preoperative assessment of laparoscopic antireflux surgery [24, 25]. In antireflux procedures for gastroesophageal reflux disease, preoperative recognition of a short esophagus is critical to a successful outcome, and thus, predictive factors of a short esophagus have been investigated by Gastal et al [24]. They asserted that only the presence of an esophageal stricture predicted esophageal shortening. Since esophageal stricture occurs after healing of acid-related inflammatory changes at the gastroesophageal junction, their results were in accord with the results of studies on acid-induced esophageal shortening or hiatal hernia development [8, 10, 24].

The pathophysiological mechanisms underlying the contribution of hiatal hernia to GERD are still not clear, although the association between hiatal hernia and reflux esophagitis has been seen to be significant, irrespective of country and race [2]. The present results also suggest a significant association between hiatal hernia and reflux esophagitis, which is in agreement with previous reports. In general, abnormal exposure of the lower esophagus to acid is an important mechanism of GERD development, and hiatal hernia has been regarded merely as a risk factor for GERD. However, the present results suggest that exposure of the esophagus to acid affects hiatal hernia, as well as GERD, development. Thus, although exposure of the esophagus to acid is considered to induce GERD directly, we propose a new pathway, in which exposure of the lower esophagus to acid initially induces hiatal hernia formation, and then some of those subjects develop GERD. Previous suggested mechanisms by which hiatal hernia could worsen acid reflux, including prolonged esophageal acid clearance, disruption of diaphragmatic sphincter function, and decreased baseline lower esophageal sphincter tone, affect the progression from hiatal hernia to GERD [3]. Furthermore, reflux esophagitis itself worsens the grade of hiatal hernia, leading to development of a vicious circle. Based on our results, which indicate that increased gastric acid secretion induces hiatal hernia development, it is possible that long-term acid suppression therapy using PPIs or PCABs, from middle age, could prevent the development of hiatal hernia to a certain extent. Although it is difficult to prove this hypothesis, since gastric acidity in the Japanese population is likely to increase dramatically because of a significant decrease in the prevalence of *H*. *pylori* infection, especially in younger generations [26], a future increase in the prevalence rate of hiatal hernia will validate our hypothesis.

In this study, there was no statistically significant association between hiatal hernia and *H*. *pylori* infection, which is generally considered to be related to gastric acid secretion. However, decreased gastric acid secretion is generally observed in *H*. *pylori* positive subjects with advanced gastric atrophy. Thus, subjects serologically positive for *H*. *pylori* infection but with normal pepsinogen status showed normal gastric juice pH (mean 1.83, data not shown) in this study, suggesting that gastric acid secretion in *H*. *pylori* positive subjects does not always decrease, resulting in a loss of the association between hiatal hernia and *H*. *pylori* infection was lost.

There are some limitations to this study. First, the study subjects were not normal, healthy subjects, and hence, selection bias should be considered. To minimize this bias, those with gastrointestinal symptoms were excluded in this study, because subjects with high acidity and hiatal hernia may frequently develop upper gastrointestinal symptoms, thus produce spurious association between high acidity and hiatal hernia. The prevalence of *H*. *pylori* infection (60.1%) in this study population (median age 65.0 years) is similar to the previously reported prevalence of *H*. *pylori* infection in the same generation [26], suggesting that the disease structure and acid secretory function of our study population are similar to those of “normal subjects”. Therefore, we believe that the etiology of hiatal hernia can be adequately analyzed using these subjects because of their similarity to the general population. Further, this study is limited by the lack of data on 24-h esophageal pH monitoring. However, because of the low acceptability and cost-ineffectiveness of 24-h esophageal pH monitoring, it is difficult to perform this kind of monitoring in a large population, as in the present study.

In conclusion, the results of the present study suggest that high gastric acid secretion is significantly associated with an increased risk of hiatal hernia. The present findings support the previously suggested notion that acid reflux contributes to causation of hiatal hernia by shortening the esophagus.

## Supporting Information

S1 Table Data analyzed. Raw data analyzed for this study.
(XLSX)
