# Correction: Association between Increased Gastric Juice Acidity and Sliding Hiatal Hernia Development in Humans

**DOI:** 10.1371/journal.pone.0172375

**Published:** 2017-02-13

**Authors:** Hiroshi Kishikawa, Kayoko Kimura, Asako Ito, Kyoko Arahata, Sakiko Takarabe, Shogo Kaida, Takanori Kanai, Soichiro Miura, Jiro Nishida

There is an error in affiliation 3 for author Soichiro Miura. Affiliation 3 should be: Department of Internal Medicine, National Defense Medical College, Tokorozawa, Saitama, Japan.
